# Country-level fire perimeter datasets (2001–2021)

**DOI:** 10.1038/s41597-022-01572-3

**Published:** 2022-07-30

**Authors:** Adam L. Mahood, Estelle J. Lindrooth, Maxwell C. Cook, Jennifer K. Balch

**Affiliations:** 1 Earth Lab, Cooperative Institute for Research in Environmental Sciences, University of Colorado Boulder, Boulder, USA; 2 Water Resources, USDA-ARS, Fort Collins, CO USA; 3 Applied Math, University of Colorado Boulder, Boulder, USA; 4 Geography, University of Colorado Boulder, Boulder, USA

**Keywords:** Natural hazards, Fire ecology

## Abstract

Fire activity is changing across many areas of the globe. Understanding how social and ecological systems respond to fire is an important topic for the coming century. But many countries do not have accessible fire history data. There are several satellite-based products available as gridded data, but these can be difficult to access and use, and require significant computational resources and time to convert into a usable product for a specific area of interest. We developed an open source software package called Fire Event Delineation for python (FIREDpy) which automatically downloads and processes all of the source files for an area of interest from the MODIS burned area product, and runs a spatiotemporal flooding algorithm that converts those hundreds of grids into a single fire perimeter shapefile. Here we present a collection of fire event perimeter datasets for every country on the globe that we created using the FIREDpy software. We will continue to improve the efficiency and flexibility of the underlying algorithm, and intend to update these datasets annually.

## Abstract (summary)

**Table:** 

Measurement(s)	Fire event occurrence • growth rate • size
Technology Type(s)	Satellite fire detections
Sample Characteristic - Environment	fire
Sample Characteristic - Location	global

## Background & Summary

Global fire activity is changing in many areas as temperatures increase and land use intensifies^1–5^. This is sparking an increase in attention given to fire activity and fire ecology. However, the availability of data for spatially delineated fire events is limited or non-existent in many countries^6^, with most global fire data coming from satellite-based active fire detections^7,8^ and gridded burned area products^9,10^. The lack of products containing delineated events has led to many global studies about fire ecology that are computationally-intensive, coarse-scale trend analyses^1,4^.

A key advantage of datasets like Monitoring Trends in Burn Severity (MTBS)^11^ or the Fire Occurrence Dataset^12^ lies in their ease of use. Since its inception in 2007 MTBS has been cited 947 times in peer-reviewed studies according to a Google Scholar search at the time of this writing, despite documented limitations for scientific use of some facets of the product^13^. The MTBS dataset is regularly updated, easy to find on the internet, and it is free, fast and easy to download and use. Many environmental scientists and resource managers do not have the computational budget or expertise in big data or remote sensing to deal with the challenges one must overcome to process large fire datasets. This is especially true for cases when all that is needed is a shapefile of fire perimeters that can be used to map fire history. Other global fire perimeter datasets have been produced from satellite-derived burned area products^14,15^, but these are only available in yearly or monthly global shapefiles. Often field-based studies of fire effects require an entire time series over study areas that are only a few hundred km in diameter^16^ or a single ecoregion^17^. The end user who wants to understand the fire history for their region would have to download yearly shapefiles with a global extent, clip all of those shapefiles to their area of interest, and then combine them into one shapefile, just to get started. We suspect that the lack of accessible fire perimeter datasets that are easy to download and use contributes to a disparity in research, where fire ecology studies are conducted mostly in developed countries that have either research infrastructure capable of handling big data or longer-term government records, or temperate forested regions that have substantial tree-ring records^18^.

**Fig. 1:**
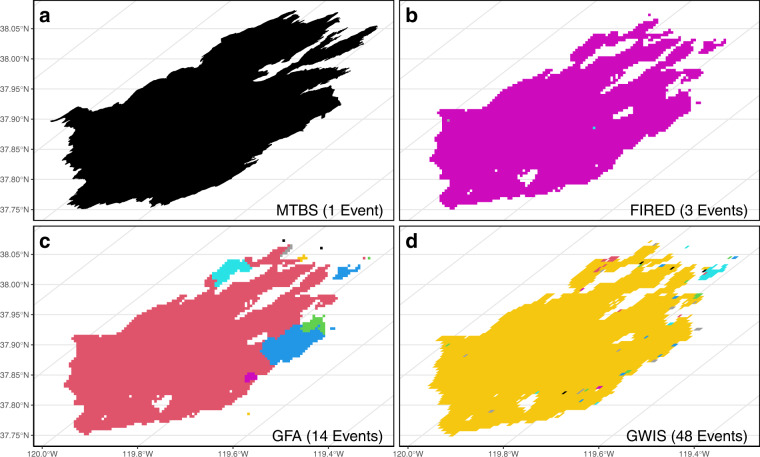
Comparison of global fire event products performance for the 2013 Rim Fire (**a**). In the FIRED product (**b**), the Rim fire was classified as one very large event with two single pixel events. The Global Fire Atlas (GFA, **c**) and Global Wildfire Information System (GWIS, **d**) each delineated a very large event, with 13 and 47 smaller events, respectively.

**Fig. 2:**
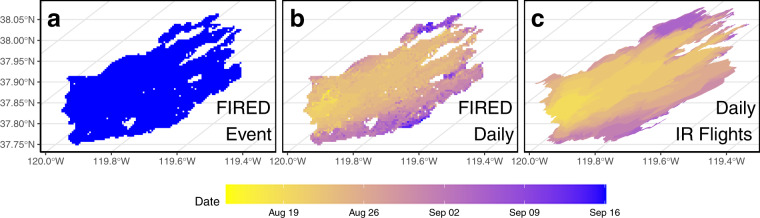
The two primary outputs FIREDpy provides are a daily- and event-level product. Panel a shows the default single event polygon. In b, each day has a separate polygon, with associated statistics generated, within each event. Panel c shows the daily perimeters derived from the airborne infrared by the incident management team for comparison.

There are two existing global perimeter products, the Global Fire Atlas (GFA) (Andela *et al*.^14^) and the Global Wildfire Information System (GWIS) (Artes *et al*.^15^). Both were created by applying spatiotemporal flooding algorithms to the MODIS MCD64 Burned Area Product. These algorithms assign burned pixels from the MCD64 products using a moving window whose size is defined by spatial and temporal parameters. They are created as monthly or yearly slices of the entire globe, and they can be subsetted. These products are extremely valuable for global scale studies. But when we look at how those products delineate known fire events we see a consistent problem in that they both seem to over-segment events in ways that appear unrealistic. This inconsistent event delineation is not problematic for coarse-scale or regional estimates of burned area or fire seasonality, but can lead to unrealistic estimates for number of fire events and event-level characteristics like fire size and spread rate. In Fig. 1 we illustrate this with an example of the 2013 Rim Fire in California, United States, which was unmistakably a single event that burned about 90,000 ha over the course of three months. Figure 2 illustrates how the day-to-day progression of the Rim Fire was a steady progression from a single ignition in late August. Table 1 shows how the differences in event delineation propagate to calculations of burned area and number of events. In the GFA, the Rim Fire is delineated as one large event of 804.5 km^2^, and 13 additional events totaling 88.7 km^2^. in GWIS it is delineated as one event of 878 km^2^ and 47 additional events totalling 20 km^2^. With FIRED, there is one event of 892 km^2^ and 2 single pixel events totalling less than one km^2^. One cause for potential differences is how one defines a “fire event”. Large fires often have multiple ignition sources. The Global Fire Atlas algorithm and others^19^, for example, search for local minima to identify various ignition locations that may begin as small patches, only to later form a large complex and in the end described with a single fire perimeter. The choice of outside sources for optimizing the spatial-temporal parameters, the method of optimization, and the intent of the final product’s meaning (defining events as single ignition patches vs contiguous burned area) all lead to different outcomes in the final events that are delineated. Another likely source of this discrepancy is that GWIS and GFA are calibrated to create a single global product. Because different geographical areas have different types of fire regimes, they have fires that grow at different rates and to different sizes, and occur in greater or fewer frequencies, and so the spatial and temporal parameters that work well for defining a fire event in one area may result in over- or under-segmentation in other areas. Here, we decided upon an approach of creating many regional products across the globe, rather than one product for everywhere on earth.

**Table 1:** Rim fire comparison.

Product	Area (km2) (Primary event)	Area (km2) (Secondary events)	Number of Secondary Events	Total Area (km2)
MTBS	1039.8	0	0	1039.8
FIRED	892.3	0.4	2	892.8
GWIS	878.8	20	47	898.8
GFA	804.5	88.7	13	893.2

**Fig. 3:**
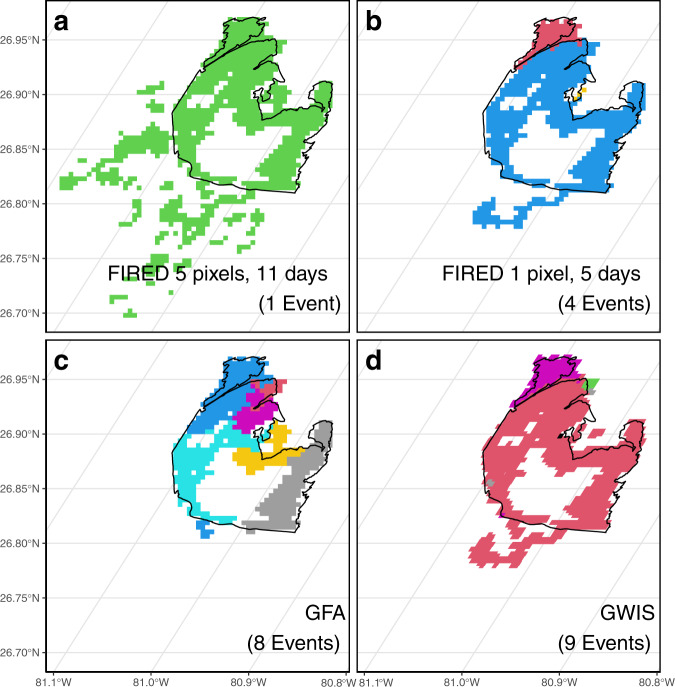
Product comparison for two small events in Florida, the Moonshine Bay and Sour Orange fires (outlined) that both ignited in February of 2007 and were delineated by MTBS. In b the firedpy product that was optimized for the entire United States with a moving window of 5 pixels, 11 days resulted in aggregation of the two fires delineated by MTBS, but also several smaller fires nearby. In b, it was re-ran with a window of one pixel and five days, for a more realistic result. Delineations by the Global Fire Atlas (**c**) and the Global Wildfire Information System (**d**) are shown for comparison.

Besides the ease of access and use, the advantage of the FIRED product lies in the user’s ability to use the open-source software, FIREDpy, to tailor the spatial and temporal parameters of the moving window algorithm in order to realistically delineate events for their region of interest. In Fig. 3, we illustrate this by comparing the three products for a pair of small fires in Florida. In this case, the FIRED product that was created with a larger moving window (5 pixels and 11 days) over-aggregated the events, but it only required one line of code at command line to recreate the product with a smaller moving window (1 pixel and 5 days) to get more realistic results.

Here, we present a collection of regionally-tailored fire perimeter datasets for every country in the world with significant fire activity^20^, which we created with the open source algorithm, FIREDpy^21^. Each dataset is either a single country or a broader region, depending on the data volume. These datasets differ from other similar efforts^14,15^ in that each dataset created by FIREDpy is a single file containing a collection of polygons that is generated for the entire time series, rather than monthly or yearly aggregations with a global extent. Furthermore, we have generated the data products at a spatial extent land managers and ecologists would typically use to do regional-scale research, and we adjusted the spatial and temporal parameters for each country to yield realistic event delineations. We also made every effort to ensure that download sizes are reasonable ( < 300 MB). They have a temporal extent from November 2000 to the summer of 2021 at the time of this writing, and they will be actively maintained and updated yearly and upon request. Most importantly the software we developed to generate the datasets is open source and freely available, and so novel fire perimeter datasets can be generated by anyone for any area of interest at any time, using a single command. We hope this will increase the capacity of ecologists and land managers across the world to study fire activity, and incorporate fire history into their work. We invite the broader community to contribute to the continued development of the software package and associated data products.

## Methods

The data were produced using an open source algorithm developed called FIREDpy: fire event delineation for python^21^. Firedpy inputs the gridded Moderate Resolution Imaging Spectroradiometer (MODIS) MCD64A1 burned area product for an area of interest, along with user-specified ancillary data (Table 2), and outputs fire perimeters with their associated characteristics (Tables 3, 4). FIREDpy is available as a software package at https://github.com/earthlab/firedpy, and the datasets we have produced are available as a data collection in the University of Colorado’s data repository^20^. Balch *et al*.^21^ previously used FIREDpy to create fire perimeters for the Coterminous United States, and here we expand upon that work by creating additional country-level products for the rest of the world.

**Table 2:** Input data.

Name	Description	Source
*MODIS MCD64A1 Burned Area*	*Monthly Gridded Burn Date Estimations (463* *m)*	^10^
*MODIS MOD12Q1 Landcover*	*Annual Gridded landcover data (463* *m)*	^25^
*CEC Ecoregions*	*Ecoregions for North America*	^23^
*WWF Ecoregions*	*World Ecoregions*	^24^

**Table 3:** List of primary variables in the FIRED datasets. All variable names are 10 characters or less in order to be compatible with the popular .shp file format.

Variable	Description	Data Type	Event, Daily or Both
id	Fire Event ID #	integer	Both
did	Day id #	character	Daily
ig_date	Date of ignition	date	Both
ig_day	Ignition day of year	integer	Both
ig_month	Ignition month	integer	Both
ig_year	Ignition year	integer	Both
last_date	last date a pixel burned	date	Both
event_dur	duration of the event	integer	Both
tot_pix	total pixels burned	integer	Both
pixels	pixel burned that day	integer	Daily
tot_ar_km2	total burned area (km2)	float	Both
dy_ar_km2	area burned that day (km2)	float	Daily
fsr_px_dy	fire spread rate (pixels per day)	float	Both
fsr_km2_dy	fire spread rate (km2 per day)	float	Both
mx_grw_px	maximum growth in pixels	float	Both
mn_grw_px	minimum growth in pixels	float	Both
mu_grw_px	mean growth in pixels	float	Both
mx_grw_km2	maximum growth in km2	float	Both
mn_grw_km2	minimum growth in km2	float	Both
mu_grw_km2	mean growth in km2	float	Both
mx_grw_dte	date of maximum growth	date	Both
ig_utm_x	coordinates of ignition	float	Event
ig_utm_y	coordinates of ignition	float	Event
tot_perim	length of the fire perimeter	float	Event
geom	geometry of the event	list of vertices	Both

**Table 4:** List of secondary variables in the FIRED datasets.

Variable	Description	Data Type	Event, Daily or Both
eco_mode	numeric ecoregion code	integer	Both
eco_name	name of ecoregion	character	Both
eco_type	ecoregion data source	character	Both
lc_mode	numeric landcover code	integer	Both
lc_name	landcover type name	character	Both
lc_type	source of landcover data	character	Both

These are generated only if explicitly specified by the user. All variable names are 10 characters or less in order to be compatible with the popular .shp file format.

**Fig. 4:**
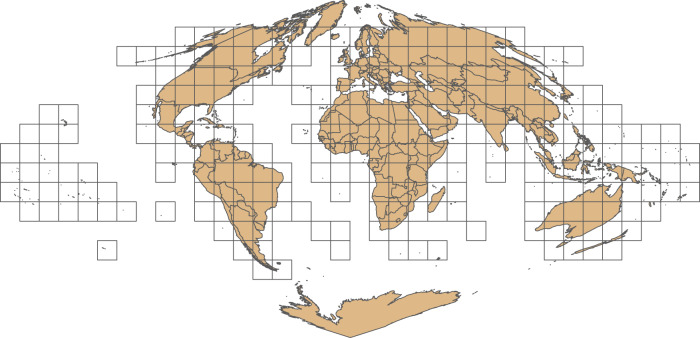
The tile system used by most MODIS products. MODIS tiles cover the entire globe, but here we show tiles that are included in the MCD64A1 burned area product.

The MODIS burned area product^10^ is a gridded product that is produced for every month of the year, starting in November 2000. It is provided to the public in monthly HDF files across a system of globally distributed tiles (Fig. 4). Each monthly HDF file contains five layers: estimated burn date, first day burned, last day burned, burn date uncertainty and a quality control layer. It is also provided in sub-continental windows and as daily shapefiles^22^.

FIREDpy is a data acquisition and processing program that is centered around a spatiotemporal flooding algorithm. It inputs a spatial parameter, temporal parameter, spatial extent and temporal extent. It uses these parameters to create a spatiotemporal moving window that assigns the same event identification number to each pixel that falls within the specified spatial distance and time period from the focal pixel. The spatial parameter is the number of 463.31271653 m pixels and the temporal parameter is the number of days. The spatial extent is an area of interest in the form of a shapefile or MODIS tile names, and the temporal extent is a start and end date. FIREDpy uses the spatial and temporal extent inputs to automatically download all of the monthly MODIS MCD64A1 hdf files within those spatial and temporal extents. It then extracts the burn date layer from each hdf file and creates a spatiotemporal cube of monthly burn dates for the entire time series for the area of interest. It uses the spatial and temporal moving window parameters to assign event identification numbers to each pixel, and then converts the pixels into polygons. The user can choose to have each event as a single polygon, or divided into separate polygons by burn date in order to see the day-to-day progression of the fire (Fig. 2). The user can also specify additional variables to be extracted to each fire event or burn date polygon, such as ecoregions^23,24^ and landcover type^25^. See Table 2 for all inputs.

For most countries, we used a spatial parameter of one pixel, and a temporal parameter of five days, following the parameters Artés *et al*.^15^ used for their global dataset. For sub-Saharan Africa and Southeast Asia (Cambodia, Vietnam, Thailand, Myanmar and Laos), we used one pixel and two days, based on the analysis of^26,27^ and after finding that using one pixel and five days in those regions tended to result in over-aggregation. For the United States and Canada we used 5 pixels and eleven days based on our own validation^21^ that agreed with other findings in temperate regions^19^. For smaller countries and regions, we produced both the daily- and event-level polygons and packaged them together in the data repository. For most areas, we only produced the event level polygons due to very large file sizes and data processing times, as the daily polygon files can be an order of magnitude larger than the event files. We encourage those who need the daily-level polygons in those areas to run the algorithm on smaller areas or time periods of interest to yield a manageable data product.

## Data Records

The firedpy github page has links to all of the datasets we have produced (https://github.com/earthlab/firedpy), which are hosted in the Earth Lab Data user collection on CU Scholar^20^. CU Scholar is an “*open access institutional repository supporting the research and teaching mission of the University of Colorado Boulder*”. On CU Scholar, each country or region is listed as FIRED followed by the name of the country or region. Each dataset is stored as a zip file that has several files inside. The first file is a readme text file that serves as the metadata. It contains a short description of the data that includes the name of all countries included, the temporal extent, the spatial and temporal parameters used for the flooding algorithm, followed by a list of the definitions of each variable that is included in the attribute table. The variables provided for the event and daily products are provided on Tables 3 and 4. The second file is the events shapefile in geopackage (.gpkg) format. Files three to six are the four files that make up an ESRI shapefile, which have the same base name and different extensions: (.shp, .dbf, .prj, .shx). The last file, if included, is a geopackage of the events split into the day to day progression. The default naming convention is fired_<AOI>_to<YYYYDOY>_<events or daily>.gpkg, where AOI is replaced by the name of the shapefile of the area of interest that is supplied as an input, YYYYDOY is the year and day of year of the end of the time series, and the last part of the base file name is whether each polygon is a whole event or day in the progression. An example for Bolivia that ends in July 2021 would be fired_bolivia_to2021182_events.gpkg.

## Technical Validation

When we created the fire perimeter datasets for the conterminous United States, we produced 225 iterations of fire perimeters—one iteration for each combination of spatial and temporal parameters between 1 and 15 pixels and days, respectively, that define the size of the moving window. We filtered the dataset to only include fires that overlapped spatially and temporally with fire events from the Monitoring Trends in Burn Severity dataset^11^. We then compared all of the iterations, and selected the spatial temporal combination (5 pixels, 11 days) that minimized both over- and under-segmentation. In other words, we aimed to find the spatial and temporal parameters that resulted in fire event perimeters that were a one to one match with the Landsat-derived MTBS perimeters. The relationship between MTBS and the spatiotemporally optimized FIRED for burned area is generally strong (R^2^ = 0.92 for all events, Balch *et al*.^21^), but it is weaker for smaller fires. Comparing among size classes revealed weaker relationships below 70,000 ha (R^2^ values between 0.5 and 0.8). Comparison with MTBS also revealed that the MODIS MCD64A1 burned area product also consistently underestimates burned area for fires below 100,000 hectares, but this is likely an artifact of MTBS not including unburned patches within its perimeters. This can be seen in Fig. 1, where the rim fire perimeter from MTBS has within its boundaries two large unburned areas. These validation methods are described in greater detail in^21^.

For the remainder of the globe we chose more restrictive combinations because we found that using 5 pixels and 11 days often resulted in over-aggregation. Since we were not able to find consistent validation data with which to optimize our parameters outside of the US, we followed the spatial and temporal parameters used by other studies that used the MODIS burned area product to delineate events (see Methods).

## Usage Notes

A typical call to the firedpy command line interface is as follows:


firedpy -spatial 1 -temporal 5 -aoi/home/firedpy/ref/individual_countries/bolivia.gpkg -shp_type gpkg


First, it specifies spatiotemporal parameters, then the area of interest (countries, continents, US states and Australian states that have been reprojected to the MODIS sinusoidal projection are provided in a reference folder) and the output shapefile type. All parameters except area of interest are set to sensible defaults (daily defaults to no, start and end date default to the entire record). The user can also simply type “firedpy”, and the software will prompt the user for the input parameters.

Running firedpy in its current form can be computationally intensive. Exactly how intensive it is depends on the spatial and temporal extents, as well as the volume of fire activity experienced by the target region. In particular it has very high requirements for random access memory (RAM). The conterminous United States, for example, can be generated on a personal computer with 16 GB of RAM. Brazil has a smaller spatial extent, but about ten times the fire activity, and needs about 100 GB of RAM to generate. Many countries in sub-Saharan Africa as well as Australia have higher resource requirements. The need for high computational requirements is one of the main reasons we generated datasets for every country, rather than simply providing the software as a tool. We are actively developing the code, and expect the software to be much more efficient in the near future. Users who do wish to create a product on their own with firedpy may wish to use the docker container we created for the purpose of running firedpy on a cloud computing platform like Cyverse (https://cyverse.org/). Instructions for using firedpy in the docker container are on the GitHub page.

## Supplementary information

Table S1 (pdf)

## Data Availability

All code is open source and available at https://github.com/earthlab/firedpy. The data we produced is available at the Earth Lab Data collection at CU Scholar^20^. Links to each dataset are provided on the front page of the aforementioned github repository, and are provided here in Table S1. The docker container with a custom software environment for running firedpy is at https://hub.docker.com/repository/docker/earthlab/firedpy.
